# The Role of the Physical and Social Environment in Observed and Self-Reported Park Use in Low-Income Neighborhoods in New York City

**DOI:** 10.3389/fpubh.2021.656988

**Published:** 2021-04-20

**Authors:** Javier E. Otero Peña, Hanish Kodali, Emily Ferris, Katarzyna Wyka, Setha Low, Kelly R. Evenson, Joan M. Dorn, Lorna E. Thorpe, Terry T. K. Huang

**Affiliations:** ^1^Department of Psychology, The Graduate Center, City University of New York, New York, NY, United States; ^2^Center for Systems and Community Design, Graduate School of Public Health and Health Policy, City University of New York, New York, NY, United States; ^3^Department of Epidemiology, Gillings School of Global Public Health, University of North Carolina-Chapel Hill, Chapel Hill, NC, United States; ^4^Department of Community Health and Social Medicine, City University of New York School of Medicine, New York, NY, United States; ^5^Department of Population Health, School of Medicine, New York University, New York, NY, United States

**Keywords:** park use, physical environment, social environment, low-income neighborhoods, built environment, community health, stop and frisk, physical activity and redesigned community spaces study

## Abstract

Physical and social environments of parks and neighborhoods influence park use, but the extent of their relative influence remains unclear. This cross-sectional study examined the relationship between the physical and social environment of parks and both observed and self-reported park use in low-income neighborhoods in New York City. We conducted community- (*n* = 54 parks) and individual-level (*n* = 904 residents) analyses. At the community level, observed park use was measured using a validated park audit tool and regressed on the number of facilities and programmed activities in parks, violent crime, stop-and-frisk incidents, and traffic accidents. At the individual level, self-reported park use was regressed on perceived park quality, crime, traffic-related walkability, park use by others, and social cohesion and trust. Data were collected in 2016–2018 and analyzed in 2019–2020. At the community level, observed park use was negatively associated with stop-and-frisk (β = −0.04; SE = 0.02; *p* < 0.05) and positively associated with the number of park facilities (β = 1.46; SE = 0.57; *p* < 0.05) and events (β = 0.16; SE = 0.16; *p* < 0.01). At the individual level, self-reported park use was positively associated with the social cohesion and trust scale (β = 0.02; SE = 0.01; *p* < 0.05). These results indicate that physical and social attributes of parks, but not perceptions of parks, were significantly associated with park use. The social environment of neighborhoods at both community and individual levels was significantly related to park use. Policies for increasing park use should focus on improving the social environment of parks and surrounding communities, not only parks' physical attributes. These findings can inform urban planning and public health interventions aimed at improving the well-being of residents in low-income communities.

## Introduction

There is increasing recognition of the contribution of parks to physical and mental health (1–16). Although there are health benefits related to living near a park (17, 18), many of these benefits involve using the park, such as the opportunity to engage in physical activity, recreation and social interactions (9, 19, 20). However, some studies have found that parks are often underused, especially in low-income neighborhoods (21–23). Thus, research is needed to better understand how attributes of parks specifically, and their surrounding neighborhoods in general, may contribute to increasing park use.

Early research on public spaces has shown that both physical and social environments influence whether people use public spaces, and that the presence of people in a place attracts others (24–26). These findings have led to ecological approaches in public health, such as the conceptual framework developed by Bedimo-Rung et al. (27), which suggests that attributes of the park environment can play an important role in park use. This model has been supported by subsequent studies showing, for example, that the number and type of facilities (physical environment) and programmed activities (social environment) in parks can be positively associated with both self-reported and observed park use (9, 23).

Beyond the park environment, physical and social aspects of the neighborhood environment have also been taken into account in previous studies. For example, studies of park use at the community level indicate that people are more likely to use a park when they do not have to cross a high-speed street to reach it (28), or when they have a safe way to cross such streets (29). A recent study found that walkability, aesthetics, and fewer physical incivilities in the area were related to increased park use in low-income neighborhoods (30). Another study identified environmental disamenities such as crime, lack of pedestrian safety and noxious land uses as barriers that affect the “social access” to parks in disadvantaged neighborhoods (31). Other studies have relied on surveys to analyze park use at the individual level. A study based on self-reported individual measures found that perceived maintenance and safety of the park were considered the highest ranked features that facilitate park-based physical activity (32). However, research findings of studies on the role of crime or perceived safety from crime on park use have been mixed (6, 10, 21, 23, 31, 33–37). Elsewhere, limited research has shown that collective efficacy may be positively associated with park use (38). Lastly, order maintenance policing measures such as Terry stops or stop-and-frisk (stops on grounds of “reasonable suspicion,” at the discretion of police officers) are known to impact the use of public spaces in low-income neighborhoods (39, 40) but have not been examined in relation to park use.

Collectively, studies to date suggest that both physical and social environments of parks and the surrounding neighborhoods contribute to park use, but the relative effect of different dimensions remains unclear. No study, to our knowledge, has examined both physical and social environments at the park and neighborhood level in one setting. In this paper, we leveraged cross-sectional data from GIS, directly observed park audits, and individual surveys from 54 low-income park neighborhoods in New York City (NYC) to address this gap. Our hypotheses were that: (1) Community-level measures of the physical and social environment of neighborhoods are associated with observed park use; and (2) The measures of individuals' perception of the physical and social environment of parks and their surrounding neighborhoods are associated with self-reported park use frequency at the individual level. We constructed and compared parallel models at the community and individual levels to explore these associations.

## Methods

### Study Sample

We used baseline data, collected between 2016 and 2018, from the Physical Activity and Redesigned Community Spaces (PARCS) Study (41). PARCS is an ongoing study on the impact of citywide park redesign and renovation on residents' well-being. Study parks were selected in neighborhoods per eligibility criteria for the New York City Department of Parks and Recreation (NYC Parks) Community Parks Initiative (42), which include higher-than-average poverty rate, growth rate, population density, and not having received capital investments in over 20 years (41). A study neighborhood was defined as a 0.3-mile Euclidean radius around each of the 54 study parks. Data were analyzed in 2019–2020.

### Study Design

Two separate models of correlates of park use were performed: Model 1 (*n* = 54 parks) consisted of a geospatial and statistical analysis of observed park use in relation to GIS measures at the community level, while Model 2 (*n* = 904) involved analysis of self-reported park use and perceived measures at the individual level. All participants were over 18 years old, lived in the study neighborhoods for at least 2 years, intended to stay in the neighborhood for at least 4 years, spoke English, Spanish or Chinese, and had no mobility limitations. The PARCS Study focused its enrollment efforts on public housing residents because low-income residents are the main target population of the Community Parks Initiative. The recruitment process and sample size estimation are detailed elsewhere (41).

The PARCS Study was approved by the City University of New York IRB (#2016-0248) and participants provided written consent prior to study enrollment.

### Outcome Variables

Park use was the outcome in both the community- and individual-level models. In the community-level model (Model 1), observed park use was operationalized using the System for Observing Play and Recreation in Communities (SOPARC) (43), a reliable park assessment tool (44). Following this method, observers were trained and visited parks a minimum of four times throughout the summer, between 9:00 a.m. and 5:00 p.m., to estimate the number of park users within a given hour. Two scans were performed at each visit, yielding a minimum of eight observations at each park. Park user counts were averaged for each park.

In the individual-level model (Model 2), self-reported park use was derived from two PARCS survey questions on park use frequency, based on a reliable instrument (2), adapted to a 30-day timeframe to reduce recall bias and to avoid overlapping the park renovation period. The first question asked how often participants visited the PARCS study-specific park in the past 30 days, while the second asked the same but in reference to parks in general. Both had the following possible answers: (1) daily, (2) 4–6 times/week, (3) 2–3 times/week, (4) once/week, (5) 2–3 times/month, (6) once/month, (7) <once/month or (8) no visit in the past 30 days. These categories were recoded into a continuous variable representing the frequency of park visits per month, by transforming each answer into a 30-day range and calculating its midpoint. This was done for both survey questions and the highest value between the two recoded continuous variables was used to represent the frequency of park use for each participant.

### Exposure Variables

Exposure variables (Table 1) were classified into two environmental facets (physical vs. social) between two geographical dimensions (park vs. neighborhood). Exposure variables in Model 1 were selected based on previous studies as parsimonious proxies of the physical and social environments of parks and neighborhoods (35). The timeframe for each of these variables corresponded to the 2-year-period prior to the SOPARC observations. Exposure variables in Model 2 were selected from survey questions of the PARCS Study to mirror as closely as possible the constructs in Model 1. These measures included questions from the Neighborhood Environment Walkability Scale (NEWS) (45), the Park Satisfaction and Perception survey (46), and the Social Cohesion and Trust score (47), which have been well-validated in prior studies on parks and health. Details of these measures are as follows.

**Table 1:** Variable categories and dimensions for Model 1 and Model 2.

**Variable**	**Model 1 (community level)^a^**		**Model 2 (individual level)^b^**	
**Dimension/Category**	**Variable**	**Source**	**Variable**	**Source**
**Park dimension**				
**Physical environment**				
	Average Park condition	NYC Parks PIP	Perceived quality of parks	Park satisfaction and perception survey
	Number of facilities	Count of children play areas + Count of athletic facilities (NYC Parks)		
**Social environment**				
	Count of events programmed in study park	Office of Citywide Event Coordination and Management	Perceived park used by others	Park satisfaction and perception survey
**Neighborhood dimension**				
**Physical environment**				
	Percentage of noxious land uses	Area of vacant lots + industrial zoning (MapPLUTO 2016 V2)	Perceived neighborhood attractiveness	NEWS survey
	Traffic accidents count	NYPD Motor Vehicle Collisions – Crashes	Perceived traffic in nearby streets	NEWS survey
**Social environment**				
	Violent crimes count	NYPD Complaint Data - Historic	Perception of crime safety	NEWS survey
	Stop-and-frisk incident count	NYPD Stop, Question & Frisk Data	Social cohesion and trust index	Collective efficacy survey
**Outcome variables**				
	Average observed park users per hour	SOPARC Observations	Self-reported park use frequency	REVAMP survey

a *Covariates: Population density (people/acre), Non-Hispanic White population (%), Female population (%), Median age (years), Mean income per capita ($), Park size (acres)*.

b Covariates: Race/ethnicity (Non-Hispanic White, Hispanic/Latino, Non-Hispanic Black, others), income ($20,000 or more, < $20,000), gender (male, female), age (years).

*NEWS, Neighborhood Environment Walkability Scale; NYC Parks, New York City Department of Parks and Recreation; NYPD, New York Police Department; PIP, Parks Inspection Program; REVAMP, Recording and Evaluating Activity in a Modified Park; SOPARC, System for Observing Play and Recreation in Communities*.

### Environmental Dimensions of Parks

In the community-level model, the physical environment variables included the average condition of a park (e.g., litter, infrastructure damage), as evaluated by the Parks Inspection Program (48), and the number of facilities in a park. The facilities count consisted of a sum of athletic facilities (49) and children's play areas (50) within a park. The social environment was represented by the number of permitted events in a park (e.g., sports tournaments, concerts), as reported by the Office of Citywide Event Coordination and Management (51).

In the individual-level model, two questions of the Park Satisfaction and Perception survey were used as measures of the park environment: “I am satisfied with the overall quality of parks in my neighborhood” was used as a proxy of the physical environment, and “Parks in my neighborhood are used by many people” was used as a proxy of the park's social environment. Both response options used a 5-point Likert scale.

### Environmental Dimensions of Neighborhoods

Model 1 used two variables as proxies of neighborhood physical environment. The first variable was adapted from Weiss et al. (31) to measure the percentage of noxious land uses (vacant lots or industrial zones). This calculation was done using QGIS 3.10, and required the MapPLUTO 2016V2 dataset from the NYC Department of City Planning (52). The second variable was the total number of traffic accidents from the “NYPD Motor Vehicle Collisions – Crashes” dataset (53). This variable served as a proxy of the neighborhood's infrastructure (e.g., roads, stop signs, pedestrian walkways) that allowed residents to walk safely.

Model 2 used three questions from the NEWS survey to assess perceived physical environment (45). “There are attractive buildings/homes in my neighborhood” served as a proxy for neighborhood attractiveness. In addition, walkability was estimated using the average score of two questions: “There is so much traffic along my street [or nearby streets] that it makes it difficult or unpleasant to walk in my neighborhood.”

For neighborhood social environment, Model 1 included total violent crime count (murder, rape, robbery, and felony assault) from the New York City Police Department (NYPD) Complaint Data – Historic dataset (54). The count of stop-and-frisk instances was also included, as reported in the NYPD Stop, Question and Frisk dataset (55), as a proxy of neighborhood social tension.

In Model 2, the question “There is a high crime rate in my neighborhood” from the NEWS survey (45) was used as a proxy for perceived safety from crime. The Social Cohesion and Trust score was used as a proxy for the degree of social harmony (47). This score, one of two components of collective efficacy, consists of five questions related to cohesion among neighbors and their willingness to act for the common good. The scale has been widely used and tested (47, 56, 57).

### Covariates

Both models controlled for age, gender, income, and ethnicity. Model 1 also controlled for park size and neighborhood-level variables: neighborhood population density, percentage of non-Hispanic Whites, percentage of female population, mean individual income, and median age. Demographics within the study areas were estimated with a Cadastral-based Expert Dasymetric System (58), using the American Community Survey 5-year estimates (2011–2016) and the MapPLUTO 2016V2 datasets in QGIS 3.10.

In Model 2, all demographic covariates, except for age, were categorical. A dichotomous question ascertained whether participants' annual household income was below or above $20,000. Ethnicity was recoded from a 16-category race variable and a binary “Hispanic” variable to form categories: “Non-Hispanic White,” “Non-Hispanic Black,” “Hispanic,” or “Other.” Non-Hispanic Whites served as the referent group.

### Statistical Analysis

Descriptive statistics were estimated for all study variables in Models 1 and 2. Multiple linear regression was used in Model 1 to determine the association of observed park use with the exposure variables mentioned above, adjusting for neighborhood covariates. In Model 2, a generalized estimating equation with negative binomial distribution was used in a sample with complete data to model self-reported park use on the exposure variables described above, adjusting for individual-level demographics and using robust standard errors to account for the effect of neighborhood clustering. Collinearity diagnostics were satisfactory in both models. All statistical analyses were performed using IBM SPSS v26 (Chicago, IL). Alpha was set at 0.05.

## Results

### Sample Characteristics

Table 2 presents the neighborhood and park characteristics for the Model 1 sample. Two study parks (3.7%) were over three acres, 23 of the parks (42.6%) were between one and three acres and 29 (53.7%) were one acre or less. The number of facilities in parks ranged from 0 to 18. Eight parks had no programmed events in the 2 years prior to park use observations; 31 had ten events or less, and the two parks with the most programmed events had 147 and 235. The population in the study areas ranged from 3,894 to 41,945 residents, with a mean (SD) of 18,371 (8,390) residents. Most residents in these study areas were low-income [mean individual income (SD) = $22,297.31 (9,648.99) per year] and either Black or Hispanic [mean (SD) = 70.7 (26.9)%].

**Table 2:** Descriptive statistics for Model 1 (community-level analysis, *n* = 54 parks).

**Variable (unit)**	**Mean (SD)**
**Demographics/covariates**	
Population in study area (estimated count)	18,370.77 (8,389.77)
Population density (people/acres)	89.82 (41.75)
Non-Hispanic White population (%)	16.34 (17.61)
Female population (%)	53.36 (3.09)
Median age (years)	34.33 (4.30)
Mean income per capita ($)	22 297.31 (9,647.99)
Park size (acres)	1.12 (0.76)
Park use (average/hour observed users per visit as observed with SOPARC)	20.91 (19.28)
**Park dimension**	
**Physical environment**	
Park facilities (count)	6.43 (3.85)
Park condition (average rating per PIP visit)	0.75 (0.21)
**Social environment**	
Programmed events in the park (count in 2 years prior to SOPARC)	18.65 (39.94)
**Neighborhood dimension**	
**Physical environment**	
Traffic accidents (count in 2 years prior to SOPARC)	747.70 (392.84)
Noxious land uses (% of study area)	6.04 (5.97)
**Social environment**	
Violent crimes (count in 2 years prior to SOPARC)	271.39 (176.23)
Stop-and-frisk incidents (count in 2 years prior to SOPARC)	241.28 (153.84)

*PIP, Parks Inspection Program; SOPARC, System for Observing Play and Recreation in Communities*.

Table 3 presents the descriptive statistics of the individual sample in Model 2. Close to half of the participants (45.8%) identified as Hispanic, 37.3% as Non-Hispanic Black, and 7.0% as Non-Hispanic White. Just over half of the participants (53.5%) reported having an annual household income under $20,000. The sample was predominantly female (79.4%), and the mean (SD) age was of 38.7 (12.1) years.

**Table 3:** Descriptive statistics for Model 2 (individual-level analysis, *n* = 904 residents).

**Variable/Category**	**Count (%)**
**Race/ethnicity**	
Non-Hispanic White	63 (6.97)
Hispanic/Latino	414 (45.80)
Non-Hispanic Black	337 (37.28)
Others	90 (9.96)
**Income**	
$20,000 or more	420 (46.46)
Less than $20,000	484 (53.54)
**Gender**	
Male	186 (20.58)
Female	718 (79.42)
	**Mean (SD)**
Age (years)	38.69 (12.06)
Park use frequency in the past 30 days (any park)	11.80 (10.11)
**Park dimension**	
**Physical environment**	
“I am satisfied with the overall quality of parks in my neighborhood”	2.02 (1.17)
**Social environment**	
“Parks in my neighborhood are used by many people”	3.06 (1.02)
**Neighborhood dimension**	
**Physical environment**	
“There is so much traffic along the street I live on [and surrounding streets], that it makes it difficult or unpleasant to walk in my neighborhood”	0.32 (1.27)
“There are attractive buildings/homes in my neighborhood”	−0.07 (1.42)
**Social environment**	
“There is a high crime rate in my neighborhood”	−0.09 (1.42)
Social cohesion and trust score	10.26 (3.46)

### Regression Models

In Model 1 (Table 4), after adjusting for covariates, average observed park use was significantly and positively associated with the number of facilities (β = 1.46; SE = 0.57; *p* < 0.05) and the number of programmed events in parks (β = 0.16; SE = 0.05; *p* < 0.01), and negatively associated with the count of stop-and-frisk incidents in the neighborhood (β = −0.04; SE = 0.02; *p* < 0.05). In other words, on average, for every 2 additional facilities available in the park, roughly 3 more people were expected to use the park per hour. For every 6 events organized in the park, one more person was expected to use the park per hour. Finally, for every 25 people stopped by the police during the previous 2 years, one less person per hour was observed using the park.

**Table 4:** Park use regressed on environmental variables at the community level (Model 1) and individual level (Model 2).

	**Model 1 (Community level)^a^**		**Model 2 (Individual level)^b^**	
**Dimension/Environment**	**Variable (unit)**	**β (SE)**	**Variable (scale range)**	**β (SE)**
**Park dimension**				
**Physical environment**				
	Park facilities (count)	1.46 (0.57)^*^	“I am satisfied with the overall quality of parks in my neighborhood” (1, 5)	−0.03 (0.03)
	Park condition (2-year average)	0.86 (10.28)	–	–
**Social environment**				
	Programmed events in the park (count)	0.16 (0.05)^**^	“Parks in my neighborhood are used by many people” (1, 5)	0.05 (0.03)
**Neighborhood dimension**				
**Physical environment**				
	Noxious land uses (%)	−0.38 (0.38)	“There are attractive buildings/homes in my neighborhood” (−2, 2)	0.02 (0.02)
	Traffic accidents (count)	0.00 (0.01)	“There is so much traffic along the street I live on [and surrounding streets], that it makes it difficult or unpleasant to walk in my neighborhood” (−2, 2)	−0.03 (0.02)
**Social environment**				
	Stop-and-frisk incidents (count)	−0.04 (0.02)^*^	Social cohesion and trust (0, 20)	0.02 (0.01)^*^
	Violent crimes (count)	0.01 (0.02)	“There is a high crime rate in my neighborhood” (−2, 2)	0.04 (0.02)

Boldface indicates statistical significance (

* p < 0.05;

** p < 0.01).

a Model 1 is a multiple linear regression on observed park use (SOPARC) adjusted for age, gender, ethnicity, income, population density and park size.

b *Model 2 is a generalized estimating equation regression with negative binomial distribution on self-reported park use frequency adjusted for age, gender, ethnicity and income*.

In Model 2 (Table 4), after adjusting for covariates, self-reported frequency of park use per month was significantly and positively associated with the Social Cohesion and Trust score (β = 0.02; SE = 0.01; *p* < 0.05). Participants who reported higher perceived social cohesion and trust among neighbors were more likely to report going to the park more often in the past month.

## Discussion

This cross-sectional study examined the relationship between the physical and social environment of parks and both observed and self-reported park use in low-income neighborhoods in New York City.

To our knowledge, this is the first study to examine the relationship between the physical and social environment of parks, using both community- and individual-level models. This study is also noteworthy given the dearth of research in low-income neighborhoods with a high proportion of Hispanics and Non-Hispanic Blacks. The multitude of neighborhood parks in NYC provided a unique opportunity to undertake this study. Furthermore, by constructing and comparing the community- and individual-level models, our study was able to operationalize and investigate potential differences or convergence in exposure-outcome relationships between objectively measured vs. self-reported or perceived variables.

Based on the results, actual physical and social attributes of parks, but not quality perceptions of parks, were significantly associated with park use. That the number of park facilities was positively associated with park use corroborates findings from prior studies (9, 33, 34). The significant relationship between programmed activities and park use is also supported by previous research (8, 9, 23, 59, 60). Together, these findings suggest that the more there is to do in a park, the more likely people will visit a park.

Although many prior studies on park use and health have focused on the importance of the physical environment (e.g., safety, cleanliness, lighting), we found that when modeled together, the social environment appeared to be more important. This was bolstered by the consistent findings in both the community- and individual-level models. The significance of social cohesion and trust is aligned with prior findings (21, 38, 57). However, to our knowledge, this is the first study to show stop-and-frisk as a significant correlate of park use. This is important because stop-and-frisk encompasses a political dimension of the social environment and is often emblematic of the deep history of racism and discriminatory practices in a context of power imbalance between those who are privileged or with authority and those who are subjugated (61–63). Therefore, in neighborhoods with high stop-and-frisk rates, the socio-political access to parks may be limited and should be further explored in prospectively designed studies. The significant stop-and-frisk finding can also be supported by studies in procedural and social justice: police harassment hinders low-income people of color's social access to public spaces (39, 40). The literature warns that the consequences of such policing practices on a neighborhood's social fabric go well beyond constraining use of public spaces; such practices can make communities of color feel dehumanized and excluded socially and spatially (39, 40, 64).

In NYC, stop-and-frisk policing was heavily criticized for disproportionately targeting low-income people of color (63), reaching a peak in 2011, when 685,724 stops were recorded; 87% of these involved Black and/or Latinx individuals and 88% were innocent (i.e., no arrests or summons issued following the stop) (65). Subsequently, the NYPD drastically reduced police stops following a court ruling (Floyd et al. v. City of New York) that deemed stop-and-frisk unconstitutional on grounds of racial profiling and discrimination (63). However, the rebranded “Stop, Question and Frisk” still disproportionately targets Black and/or Latinx population: out of 13,459 stops in 2019, 88% were made to people in either of these minority groups (63, 65). In the context of our study, this translated into lower park use among minority low-income populations in NYC. We chose to refer to the practice as stop-and-frisk throughout this paper because that is how the incidents are referred to within the NYPD dataset, and also to emphasize that this type of policing is still problematic, regardless of its rebranding and significant drop in the frequency of stops. Policing practices similar to stop-and-frisk occur in other cities as well, but not all cities publicly share stops data like NYC does, so it can be harder to identify discriminatory practices and study the associations between policing and park use (66).

Another interesting finding of our study was the non-significance of the actual crime and perception of crime variables in the models. Given the importance that some previous studies have attributed to perception of crime as a deterrent of park use (4, 34, 67), a significant relationship might have been expected, especially in the individual-level analysis, as our study had a majority of female respondents and it has been found that women have a higher likelihood of feeling unsafe in public spaces (68). However, as studies have shown mixed results regarding the role of crime in park use (33, 69), and because of the relationship between design features of a park and perceptions of safety (70), more research is warranted to elucidate the circumstances in which crime may be a significant factor. NYC is a highly urban and dense with relatively low crime per capita compared to many other cities. Thus, the role of crime or its perception in park use may be different than elsewhere. The lack of association of the traffic/walkability variables is also surprising, especially since a recent study found pedestrian safety was correlated with more children using parks in NYC (71). It is possible that this factor is more important among children, particularly given the dense and generally highly walkable urban design in a city such as New York.

Despite the study results, we cannot fully conclude that the perceived physical environment of a park is unrelated to park use. It is important to keep in mind that all parks in this study had not received capital funding in at least the past 20 years. Parks with more recent investment might show different results. In a more heterogeneous sample of parks, it is possible that there would be a stronger relationship between perceived physical park environment and park use, but further studies are needed to confirm this.

### Limitations

There are several limitations in this study. First, this was a cross-sectional study so no causal relations could be inferred. Second, this study was not meant to comprehensively capture all aspects of the physical or social environment of parks or neighborhoods. Our goal was to construct parsimonious models that could contrast the two environmental and two geographical aspects at community and individual levels in parallel. We sought to include proxy variables with an empirical basis in the literature. Other variables have been identified in the literature (e.g., relaxing atmosphere, shady trees, advertising), but were not included in our study, both because we had no reliable sources of data for our sample, and because the sample of 54 parks in our community-level analysis limited the number of variables we could add to the model. Future studies could build on the present study to design a comprehensive model of environmental correlates of park use and include other variables that we did not take into account. Finally, our study may not be generalizable to other cities, given the relatively unique urban and cultural landscape of NYC.

## Conclusions

As cities increasingly adopt policies to invest in parks and public spaces, it is important to consider that the social environment, including social interaction, cohesion and trust, may be just as, if not more, important than the physical design of such places. This is an area that neither the urban planning nor public health fields has emphasized to date. To foster park use, this study supports prior research on the importance of increasing resources for programmed activities in parks. Furthermore, local policies that affect neighborhood social relationships, such as policing practices, should also be considered and factored into the design of parks. In turn, increased park use may contribute new opportunities for social interaction and for fostering cohesion among residents. However, if social and political access to parks is not guaranteed and enhanced, these benefits may never be realized. This study, given its unique methodological design and focus on low-income, minority-majority communities, makes an important contribution to our understanding of how different dimensions and scales of the built and social environment play a role in park use. Findings of this study can inform urban planning and public health policies and interventions aimed at improving the well-being of residents in underserved communities.

## Data Availability Statement

The datasets generated for this article are not readily available because they form part of an ongoing study. Requests to access the datasets should be directed to terry.huang@sph.cuny.edu.

## Ethics Statement

The studies involving human participants were reviewed and approved by the City University of New York Institutional Review Board (#2016-0248). The patients/participants provided their written informed consent to participate in this study.

## Author Contributions

JO: conceptualization, methodology, software, formal analysis, investigation, data curation, writing – original draft, and visualization. HK: methodology, software, validation, formal analysis, data curation, and visualization. EF: data curation, writing – review and editing, and project administration. KW: methodology, validation, formal analysis, data curation, and writing – review and editing. SL: conceptualization and writing – review and editing. KE, JD, and LT: writing – review and editing. TH: conceptualization, methodology, validation, investigation, resources, writing – review and editing, supervision, and funding acquisition. All authors contributed to the article and approved the submitted version.

## Conflict of Interest

The authors declare that the research was conducted in the absence of any commercial or financial relationships that could be construed as a potential conflict of interest.

## Abbreviations

NEWS: Neighborhood Environment Walkability Scale
NYC: New York City
NYC Parks: New York City Department of Parks and Recreation
NYPD: New York Police Department
PARCS Study: Physical Activity and Redesigned Community Spaces Study
SOPARC: System for Observing Play and Recreation in Communities.
